# Rise of Raman spectroscopy in neurosurgery: a review

**DOI:** 10.1117/1.JBO.25.5.050901

**Published:** 2020-05-01

**Authors:** Damon DePaoli, Émile Lemoine, Katherine Ember, Martin Parent, Michel Prud’homme, Léo Cantin, Kevin Petrecca, Frédéric Leblond, Daniel C. Côté

**Affiliations:** a Université Laval, CERVO Brain Research Center, Québec, Canada; b Université Laval, Centre d’optique, Photonique et Lasers, Québec, Canada; c Polytechnique Montréal, Department of Engineering Physics, Montréal, Canada; d Centre de Recherche du Centre Hospitalier de l’Université de Montréal, Montréal, Canada; e Hôpital de l’Enfant-Jésus, Department of Neurosurgery, Québec, Canada; f McGill University, Montreal Neurological Institute-Hospital, Department of Neurology and Neurosurgery, Montreal, Canada

**Keywords:** Raman, coherent anti-Stokes Raman scattering, stimulated Raman scattering, neurosurgery

## Abstract

**Significance:** Although the clinical potential for Raman spectroscopy (RS) has been anticipated for decades, it has only recently been used in neurosurgery. Still, few devices have succeeded in making their way into the operating room. With recent technological advancements, however, vibrational sensing is poised to be a revolutionary tool for neurosurgeons.

**Aim:** We give a summary of neurosurgical workflows and key translational milestones of RS in clinical use and provide the optics and data science background required to implement such devices.

**Approach:** We performed an extensive review of the literature, with a specific emphasis on research that aims to build Raman systems suited for a neurosurgical setting.

**Results:** The main translatable interest in Raman sensing rests in its capacity to yield label-free molecular information from tissue intraoperatively. Systems that have proven usable in the clinical setting are ergonomic, have a short integration time, and can acquire high-quality signal even in suboptimal conditions. Moreover, because of the complex microenvironment of brain tissue, data analysis is now recognized as a critical step in achieving high performance Raman-based sensing.

**Conclusions:** The next generation of Raman-based devices are making their way into operating rooms and their clinical translation requires close collaboration between physicians, engineers, and data scientists.

## 1 Introduction

Neurosurgery can be used to treat a multitude of disorders ranging from brain tumors and cancers to traumatic brain injury, epilepsy, and Parkinson’s disease (PD). 13.8 million neurosurgical procedures are carried out worldwide every year, and it is estimated that an additional 5 million neurosurgical conditions go untreated annually.^1^ Neurosurgeons face many challenges that are unmet by modern surgical techniques: incomplete tumor resection, inaccurate surgical guidance, expensive and inefficient intraoperative diagnostics, and a relatively high risk of adverse events.

Optical technologies have gained considerable traction in neurosurgery over the last few decades. The use of 5-aminolevulinic acid-induced cancer fluorescence in glioblastoma surgery for margin detection has become common.^2^^,^^3^ More recently, a variety of other techniques are being investigated for tumor margin detection including quantitative exogenous fluorescence,^4^^,^^5^ endogenous fluorescence lifetime imaging,^6^^–^^8^ optical coherence tomography (OCT),^9^ hyperspectral imaging,^10^ and Raman spectroscopy (RS).^11^ Optical techniques are also showing promise when applied to other neurosurgical procedures. For example, deep brain stimulation (DBS) for PD could be optically guided using laser Doppler flow (LDF) measurements,^12^ diffuse reflectance spectroscopy (DRS),^13^^–^^15^ and coherent Raman (CR) spectroscopy.^16^ During epilepsy surgery, hyperspectral imaging is being investigated to help guide resection. ^17^ Finally, in closed biopsies, OCT has been used to image blood vessels to minimize hemorrhage rates^18^ and RS has shown promise in effective tumor targeting.^19^^,^^20^

Here we focus on the potential of RS to improve several neurosurgical workflows. RS is an advantageous modality for biomedical applications because it can provide label-free, molecular-specific information from tissue within safe limits of optical power. By analyzing this information with effective data science models, RS can be used to provide real-time discriminatory feedback and guidance to neurosurgeons. RS-based tools could be used to discriminate tumor and nontumor tissue for cancer resection, to detect blood vessels for safe biopsy acquisition, and to detect novel biomarkers for disease diagnosis. However, there remain a number of challenges when applying RS clinically. The Raman scattering effect is a weak phenomenon; interference from other optical processes can affect the measurable signal. Moreover, analysis of large spectral datasets obtained from complex biological mixtures can be a daunting and time-consuming task. Herein, we provide an outline of RS utility during neurosurgical procedures, a summary of spontaneous and coherent RS techniques, and a thorough review of the leading-edge systems and data analysis techniques already being deployed in, or in development for, a neurosurgical setting. All of this is provided so that future endeavors in this arena can be undertaken with clear objectives.

## 2 Clinical Challenges in Neurosurgery

The primary objectives of neurosurgeries involving brain tumors are (1) procuring quality biopsy tissue for accurate diagnosis and (2) achieving maximal cancer resection while minimizing injury to the normal brain.^21^^,^^22^ Extensive multimodal imaging [e.g., magnetic resonance imaging (MRI), x-ray computed tomography (CT) scans, and positron emission tomography scans] is used preoperatively to characterize the location of the tumor, its relationship within the brain, and the imaging features of the tumor. These images are also used by neuronavigation tracking devices during surgery. However, due to poor resolution and sensitivity, none of the preoperative imaging techniques can visualize the full extent of invasive brain cancer, limiting surgical planning. In addition, neuronavigation is highly susceptible to the shifting of the brain once the dura has been opened and tumor and cerebrospinal fluid are removed.^23^^,^^24^

For certain brain lesions, a closed biopsy for tissue diagnosis is most appropriate. Studies have shown that brain biopsies may be nondiagnostic in up to 10% of cases, and even if a diagnosis is achieved, it is found to be inexact in as many as 23% of cases.^25^

In functional neurosurgery, the aim is to relieve patients suffering from chronic neurological or neurodegenerative disorders such as PD, chronic pain, epilepsy, or dystonia. DBS consists of the surgical implantation of electrodes deep inside the brain where they modulate specific brain nuclei to correct dysfunctional brain circuits. Although many conditions can benefit from DBS surgery, its most common application is for alleviating motor symptoms of PD. The main challenge of DBS in this application is the accurate positioning of the electrode inside the target nuclei.^26^ Again, electrode placement accuracy is dictated by the quality of the preoperative imaging used and the precision of the neuronavigation tracking devices.

RS has shown great promise in overcoming these challenges. In brain tumor surgery, it provides real-time, molecularly specific information to neurosurgeons, accurately predicting the nature of the probed tissue prior to its resection. For closed brain biopsies, it can increase diagnostic yield and minimize harm by targeting cancer tissue before the biopsy sample is harvested. And, in functional neurosurgery, RS can guide electrode placement to improve safety, accuracy, and clinical outcomes. RS is only beginning its utility in neurosurgery compared with other domains. Neurosurgeries are relatively rare and less accessible than procedures for breast, lung, and gastro-intestinal oncology.^1^^,^^27^ Moreover, there is no room for error in brain tissue: minor inaccuracies might translate to major deficits for the patient. Therefore, in neurosurgery, the clinical translation of a new modality requires a particularly high confidence in its safety and clinical utility.

## 3 Raman Spectroscopy Techniques: A Short Primer

Although it is not the objective of this review to provide theory on the Raman effect, it is important to have an idea of the signal generation with respect to the sample and the optical power used. Furthermore, the wavelength of the generated signal can be an important design factor given operating room lighting, microscope illumination, and background signals. Finally, spectral content is a critical aspect of the diagnostic ability associated with a clinical system and so the techniques for spectral acquisition are also discussed. For more complete details of Raman theory, abundant resources exist.^28^^–^^31^ The modalities that have been used in neurosurgical studies include spontaneous Raman (SR) scattering, coherent anti-Stokes Raman scattering (CARS), and stimulated Raman scattering (SRS).

### 3.1 Spontaneous Raman Scattering

Since its discovery by Sir C. V. Raman in 1928, Raman scattering of light has been widely adopted as a molecular probing tool in the fields of biology and chemistry. The main feature of RS is the concept of vibrational energy: molecules in a sample vibrate. At room temperature, most molecules are in their ground state, i.e., the lowest energy level. When excited by electromagnetic radiation, the molecules will either absorb or scatter the excitation photons. Most of the scattering is elastic (Rayleigh scattering) in which the molecule is transiently raised to a virtual energy level by a photon of specific energy and almost immediately returns to the ground state by emitting a photon of the same energy. In approximately one out of 10 million scattering events, however, the energy of the emitted photons will have changed relative to the incident light. This phenomenon is called the Raman effect or inelastic scattering. Inelastically scattered photons that are of lower energy than the exciting light source is known as Stokes scattering. Scattered photons that gain energy relative to the incident light is known as anti-Stokes scattering. The Raman spectrum is a mapping of the intensity of scattered light as a function of its shift in frequency or Raman shift. It is in essence a vibrational profile of the molecules present in the interrogated sample subject to the partial volume effect.^32^

In SR, the detected spectra are a linear combination of signals from all of the molecules in the illuminated sample. Furthermore, the Raman intensity increases linearly as a function of excitation power and exposure time, facilitating spectral analysis. SR suffers from two limitations in biological tissue: low Raman signal (in absolute terms and relative to background) and high contamination from autofluorescence (AF) signals from tissue and instrument components (i.e., fiber optics). Most vibrational spectroscopy techniques aim to overcome these challenges.^33^

### 3.2 Coherent Anti-Stokes Raman Scattering

CARS is a multiphoton spectroscopy technique that uses two excitation wavelengths—a “pump” and a “Stokes” beam. When the difference between the two excitation frequencies is equivalent to a target vibrational mode, resonance occurs, generating strong nonlinear anti-Stokes signal. Although it is less practical as a means to acquire data at macroscopic scales, CARS has several advantages over SR imaging: (1) it can be more sensitive to a specific vibration, (2) it provides intrinsic optical sectioning due to nonlinear signal generation, and (3) signal generation is blue-shifted, removing the need for single-photon AF removal.^34^

In 1999, Zumbusch from the Xie group^35^ revived CARS imaging for biological tissues. Subsequently, groups continued to apply CARS to a multitude of biological structures in which it provided contrast from vibrations in DNA, lipids, proteins, and water.^36^^–^^39^ In 2005, Wang et al.^40^ presented the first imaging of myelin in *ex vivo* guinea pig spinal cord. Soon after, the landmark study by Evans et al.^41^ reported CARS imaging of *in vivo* tissue at video rate speeds, and since then, many others have followed in refining and optimizing CARS for *in vivo* imaging of nervous tissue.^42^^–^^47^

The signal generation for CARS is proportional to the quadratic intensity of the pump field multiplied by the Stokes field. CARS also scales quadratically with the number of oscillators in the sampled volume, making it specifically useful for interrogating high-density substances. In brain tissue, myelin (wrapped around many axons) is the main contrast agent due to its abundance of ${\mathrm{CH}}_{2}$ moieties.

Although CR scattering is generated at both Stokes (coherent Stokes Raman scattering, CSRS) and anti-Stokes (CARS) frequencies, the anti-Stokes signal is more commonly detected as it is stronger and unaffected by AF.

The main source of background signal in CARS is known as the nonresonant background; it is independent of the Raman shift and the excitation wavelength. Due to this effect, CARS is limited in sensitivity to sensing only high concentrations of molecules, in contrast to SRS which is unaffected by nonresonant background sources.

### 3.3 Stimulated Raman Scattering

Just under a decade after the Xie group introduced CARS to biological imaging in 2008, Freudiger et al.^48^ of the same group presented fast stimulated Raman spectroscopy (SRS) imaging of biological tissue *in vivo*. Although this was not the first use of SRS in microscopy, it was the first study to use optical powers that were safe for live animal imaging, enabled by phase-sensitive lock-in detection. The potential for SRS was rapidly demonstrated by several groups, displaying an ability to image DNA mitosis,^49^ protein dynamics,^50^ and even measure neurotransmitter concentrations.^51^ Due to its increased sensitivity over CARS for molecules at low concentrations, SRS is ideal for imaging nuclear contrast in neuropathology.^52^^,^^53^

As for CARS, the signal generation for SRS is nonlinear. However, the SRS signal is proportional to the product of the intensity from the pump field and the Stokes field. Furthermore, unlike CARS, SRS has a linear dependence with oscillators density, making the detected signal easier to correlate to molecular concentrations within the sample. The wavelength of signal generation for SRS can correspond to a stimulated Raman gain or a stimulated Raman loss depending on the frequency of the probe (i.e., which is modulated for detection). Laser noise, shot noise, and electronic noise are all sources of noise in SRS imaging. Minimizing them is especially important as small changes in the excitation laser must be measured due to the modulated aspect of SRS. The main sources of background for SRS are Raman-independent pump–probe effects. These include transient absorption, cross-phase modulation, and photothermal effects, all of which are negligible in CARS.^24^ A theoretical review of the signal-to-noise-ratio (SNR) for CR techniques is provided by Min et al.^30^

### 3.4 Spectral Imaging with Coherent Raman Techniques

CR imaging has historically been used for single-frequency imaging, creating contrast from the coherent vibration of only a few molecular bonds. However, this is limited in spectral information and therefore in diagnostic capability. In an effort to achieve rapid hyperspectral image acquisition, strategies to produce CR spectra have been developed. These techniques and their implementations are reviewed in detail by Alfonso-Garcia et al.^29^ Briefly, such strategies include the followings.

1) Multiplex or broadband CR in which one narrowband pulse and one broadband pulse are combined to create a simultaneous spectrum.
2) Spectral focusing wherein spectrally chirped pump and Stokes pulses are temporally swept, creating complete Raman shift spectra.
3) Temporal sweeping of two replicas of a broadband pulse, resulting in temporal interferences.

The technique deployed for hyperspectral CR imaging is outside the scope of this review; however, in designing a clinical system, the economic impact and technical difficulty of its implementation should be considered.

## 4 Spectroscopy Systems for Tissue Characterization in Neurosurgery

In recent years, both SR and CR have shown potential for improving safety, accuracy, and extent of resection for neurosurgical procedures. In SR, more readily available micro-optical components and more sensitive detectors have greatly increased probe efficiencies. Now, even small form-factor probes are able to detect enough SR signal for high-accuracy tissue classification at clinically relevant acquisition speeds, leading to a surge in clinical translation. For CR, the clinical adoption can primarily be traced back to breakthroughs in compact pulsed fiber-laser sources. The drastic decrease in laser size and increase in robustness has allowed for the development of portable CR microscopy systems capable of being transported into the operating room on a single cart.^54^

The exploitation of the Raman effect in neurosurgery can be divided into three main system types: (1) single-point RS probes for intact tissue assessment (mainly SR systems); (2) portable Raman microscopes for rapid histopathological evaluation after tissue resection (mainly CR systems); and (3) endoscopic imagers for intact tissue histopathology and surgical guidance (SR and CR prototypes). This section provides an overview of the hardware and technical considerations required for clinical implementation of these systems.

### 4.1 Intact Brain Tissue Interrogation Using Point Probes

RS does not require sample preparation and is thus able to interrogate intact and unlabeled tissue. Although there have been many pioneering studies in the field of neuroscience using RS, few have bridged the gap from fundamental research to clinical utility. The first *in vivo* RS used in brain cancer patients was reported in 2015. *In vivo* implementation of CR imaging in the human brain has not yet been reported. Tables 1 and 2 summarize the relevant clinical work using point probe systems; they will also be briefly described here.

**Table 1:** Optical characteristics of neurosurgical point probes.

References	Specimen	*In vivo*	Modality	Raman excitation wavelength (nm)	Raman shift (${\mathrm{cm}}^{-1}$)	Excitation spot size (mm)	Avg. optical power (mW)	Irradiation ($W/{\mathrm{cm}}^{2}$)	Acquisition time (s)	Radiant exposure single acquisition ($J/{\mathrm{cm}}^{2}$)	Spatial res. (mm)	Spectral res. (${\mathrm{cm}}^{-1}$)
2007^55^	Porcine	No	SR	719	2400 to 3800	0.3	80	113	10	1131	∼1	8.0
2010^56^	Mouse	Yes	SR	830	900 to 1800	0.4	120	95	5	477	∼1	4.0
2015^11^	Human	Yes	SR	785	381 to 1653	0.5	37–64	33	0.15	4.9	∼1	1.8
2017^57^	Human	Yes	SR, (AF, DRS)	785	381 to 1653	0.5	27–75	38	0.15	5.7	∼1	1.8
2018^16^	Macaque	No	CR	792, 1030–1044	2800 to 3050	0.01	80	$1.0\times 10^{5}$	0.001×λ	1019^a^	<0.01	1.0
2018^19^	Human	Yes	SR	671	2800 to 3600	0.35	10	10	2.0–6.0	31^b^	∼1	1.8
2019^20^	Human	Yes	SR	671, 785	400 to 1800, 2800 to 3600	0.35	20	21	1.1–6.3	62^b^	∼1	1.8

Note: Avg., average and Res., resolution.

a Number of wavelengths: 10, corresponding to Ref. 16.

b Average of 3-s acquisition time.^19^^,^^20^ Irradiance (I) is calculated using the equation: $I=P_{\mathrm{Avg}}\times A$, where $P_{\mathrm{Avg}}$ is the average power and A is the area of the beam spot on the tissue. The higher limit of the average power range is used for each case. Radiant exposure (H) is calculated using the equation: H=I×t, where I is the irradiance and t is the acquisition time (i.e., the time of light exposure.)

**Table 2:** System characteristics of neurosurgical point probes.

Year	Probe encasement	Probe OD (mm)	Autoclave ready	Optical setup	Detector	Optical transport	Probe description	Purpose
2007^55^	None	0.33	Yes	Uncased probe free space optics	−70°C cooled CCD (Renishaw)	Silica	1×300 μm MM source	Research probe for brain tissue classification in neurosurgical guidance
							Same fiber collection	
							No filters	
2010^56^	Details N/A (Sedi-ATI)	1.6	Yes	All fibered system on cart	Details N/A, CCD (Horiba)	Silica	1×400 μm MM source	Stereotactic probe for tumor delineation in mouse cancer models
							9×200 μm MM collectors	
							No filters	
							Lower AF due to 830 excitation	
2015^11^	Stainless steel needle type (EmVision LLC)	2	Yes	All fibered system on cart	−40°C cooled CCD (Andor)	Silica	1×200 μm MM source	Handheld probe for tumor delineation and guided resection
							7×300 μm MM collectors	
							Micro-optic emission filter	
							Micro-optic collection filter	
							Custom lens	
2017^57^	Stainless steel needle tube (EmVision LLC)	2	Yes	All fibered System on cart	−40°C cooled CCD (Andor)	Silica	1×272 μm MM Raman source	Multimodal handheld probe for tumor delineation and guided resection for improved cancer classification
							7×300 μm MM collectors	
							1×300 μm MM AF/DRS source	
							1×300 μm MM AF/DRS collector	
							Micro-optic emission filter	
							Micro-optic collection filter	
							Custom lens	
2018^16^	Stainless steel needle tube (in-house)	0.4	Yes	All fibered System on cart	Photon counter (H8259-02, Hamamatsu)	Silica	1×125 μm SM source	Modified DBS stylet for electrode implantation trajectory measurements in macaque cortex
							1×125 μm MM collector	
							Optional gradient index (GRIN) lens for distal focusing	
2018^19^	Modified biopsy canula (Medtronic, Inc.)	2	Yes	All fibered System on cart	−40°C Cooled CCD (Andor)	Silica	1×125 μm MM source/collector	Modified biopsy needle canula housing fiber-optic probe for biopsy guidance
							Angle polish for side viewing	
							No micro-optical components required	
2019^20^	Modified biopsy mandarin (EmVision LLC)	0.9	Yes	All fibered System on cart	−80°C cooled CCD (Andor)	Silica	1×100 μm MM source	Modified biopsy needle mandarin housing fiber-optic probe for biopsy guidance
							12×100 μm MM collectors	
							Micro-optic emission filter	
							Micro-optic collection filter	
							Prism for side viewing	

Note: Abbreviations: OD, outer diameter; SM, singlemode; MM, multimode; and N/A, not available.

In 2005, Santos et al.^58^ from the Puppels group first implemented high-wavenumber (HWN) RS for fiber-optic brain tissue sensing. By measuring only the Raman shift distant from the excitation wavelength, they showed that the probe needed no distal optics to remove contaminating Raman or fluorescence background from the silica fibers. They followed this with two more studies with and without the fiber-optic probe in *ex vivo* porcine brain tissue, demonstrating the ability of HWN RS to classify brain regions.^55^^,^^59^

Another system for brain tissue sensing with an SR probe was reported by Beljebbar et al.^56^ in 2010, which included an analysis of Raman spectra taken from an *in vivo* mouse model of glioblastoma. The probe was compact, designed professionally (SEDI, France) and acquired data in the fingerprint region. For reference, there has been extensive work in the biomedical optics field to develop optimal probes for spectroscopic sensing.^60^^–^^62^

**Fig. 1:**
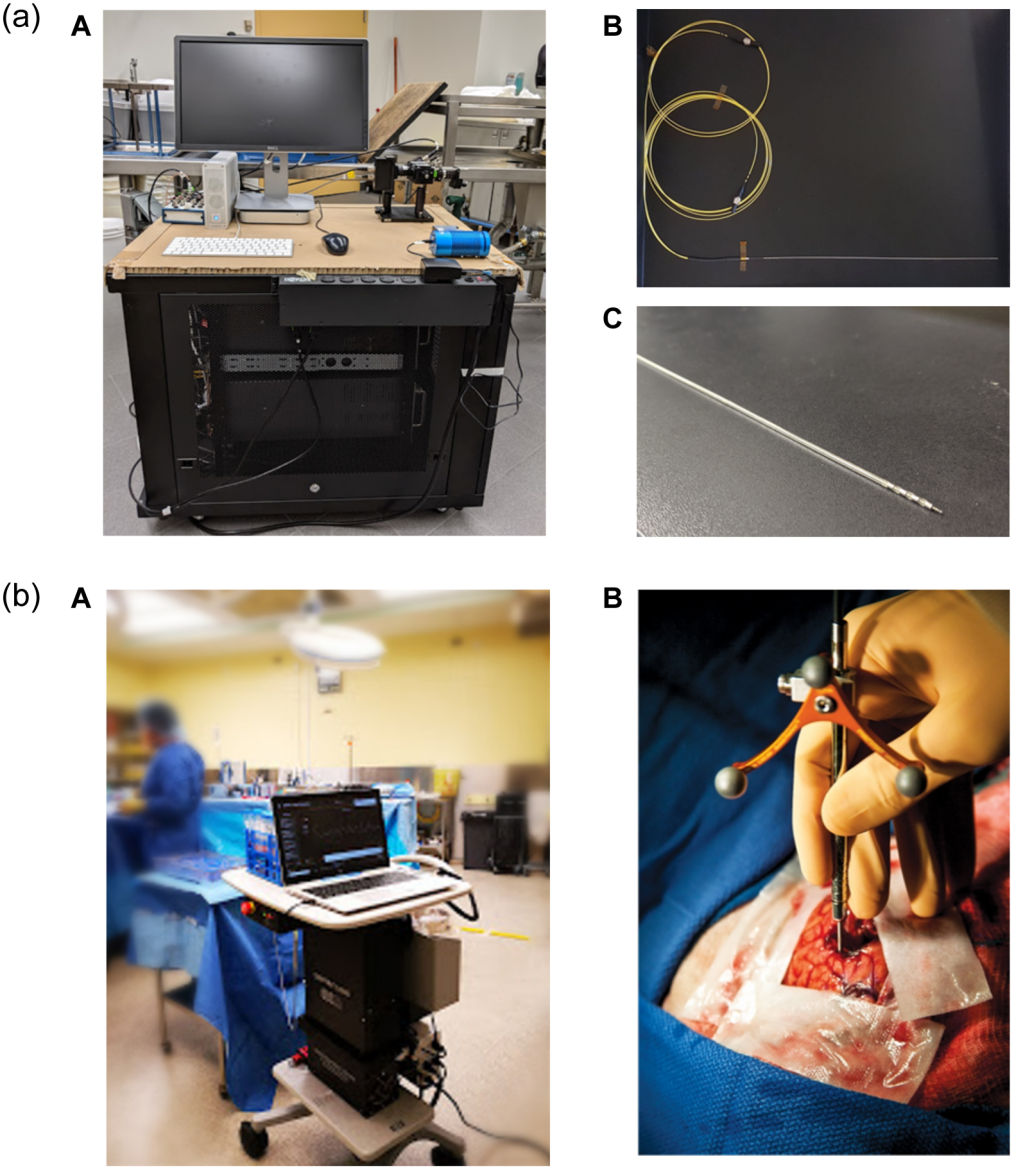
Stages of translational systems for neurosurgery. (a) Preclinical and research-oriented system for *ex vivo* human surgery. Presented here is a portable CARS spectroscopy system for performing optical measurements during a DBS electrode implantation in human cadavers (Côté lab). The system consists of (A) an encased fibered laser source, an external photon counting detector, and a computer for processing. The optical probe is inserted within the DBS electrode as shown in (B); however, the tip of the electrode has been cut off to allow the probe to be in contact with the tissue (C). (b) Clinical and commercial system for *in vivo* human surgery. Presented here is a handheld contact fiber-optic probe for SR spectroscopy, commercialized by the company ODS Medical. The system consists of (A) a 785-nm laser, a high-resolution CCD spectroscopic detector, and a computer for processing, as well as (B) a professionally designed and sterilizable contact probe for clinical use. The probe is used to interrogate live brain tissue during neurosurgery as shown in (B).

An important advancement of Raman systems in neurosurgery was presented by Jermyn et al.^11^ in 2015, marking the first use of RS in living human brain tissue. They succeeded in acquiring SR spectra from glioma patients in the operating room, and the system successfully discriminated normal brain tissue from cancer with 90% accuracy. Much of the system’s success was enabled by a professionally designed optical probe (EmVision LLC) to maximize photon collection and high-level data analysis procedures. Moreover, the design of the probe facilitated clinical use: it was hand-held, ergonomic, and durable and had a flexible fiber. Furthermore, the probe used neuronavigation markers on the back-end to register locations of measurements in the surgical planning suite [Fig. 1(b)].

Since then, the same group has pushed for clinical translation of this system. They have better characterized the system’s operating conditions in the operating room,^63^ improved data analysis to minimize ambient light contributions,^64^ and compared tumor margin localization between MRI and SR.^65^ The system has been commercialized [ODS Medical, Fig. 1(b)] and is currently in clinical trials to quantify the clinical improvement of its use.

In 2017, a similar probe was presented with multiple modalities allowing it to perform SR, AF, and DRS detection for increased classification accuracy.^57^ This system used a different data analysis procedure and was translatable to other cancers (colon, melanoma, and lung). In 2018, Desroches et al. followed up on the work by the Puppels group by using only the HWN region to interrogate human brain tissue intraoperativeley.^13^^,^^58^ Probes were used in both *ex vivo* porcine tissue and *in vivo* human tissue, with the goal of being a proof-of-principle device for HWN tumor classification. In the HWN setup, the Raman background from silica is minimal, negating the requirement for filters at the probe tip and facilitating probe miniaturization.

The next intraoperative SR milestone was spectral acquisition from deep within the brain by Desroches et al. in 2019 [Fig. 1(b)].^20^ Here the mandrin of a biopsy needle was replaced by a probe (EmVision LLC) that was optically similar to that used by Jermyn et al. in 2015, with the significant differences being its smaller size and its angle-facing detection.^11^ The probe had some spectral discrepancies from the original, probably due to the magnesium fluoride prism used for side reflection. Although this work was preliminary, it did present the first intraoperative deep brain SR measurements (both fingerprint and HWN) and opened the door for Raman-guided biopsy sampling. A variety of other probes have been implemented on *ex vivo* tissue. In 2016, Stevens et al.^66^ presented a probe design that used a collimated beam through an empty biopsy needle to measure signal from *ex vivo* porcine tissue. This probe is unlikely to be brought into the clinic in its current form due to the free-space optics involved and the long integration times required. However, it succeeded in acquiring the signal from low wavenumbers below $700\;{\mathrm{cm}}^{-1}$—routinely ignored due to the background signal from silica.

In 2018, DePaoli et al.^16^ presented a CR probe to investigate ex vivo primate brain tissue using a previously designed wavelength-sweeping system.^45^ The system was composed of a compact fiber-based pulsed laser source [Halifax Biomedical, Fig. 1(a)], a sensitive photon counting detector, and traditional silica fiber-optic probes. Rather than being measured using a spectrometer, the spectra were encoded in time using the fast wavelength-tuning lasers. The major implication of this system was the short integration time required (10 ms for low-resolution HWN spectra) given the small size of the probe. Importantly, the probe’s form factor allows it to be placed within a DBS electrode hollow core for functional neurosurgery guidance [Fig. 1(a)]. However, more work is required to minimize the optical irradiance in brain tissue before iterations of this system can be used in humans. Technical information about the systems mentioned in this section can be found in Tables 1 and 2.

### 4.2 Strengths and Limitations of *in situ* Raman Spectroscopy in Neurosurgery: Spontaneous versus Coherent

There are currently a number of advantages of SR systems over CR systems for clinical spectroscopy use. Specifically, since SR is a linear process, it allows for several leniencies in the system design, such as:

1) The use of a continuous laser source rather than a pulsed laser, allowing for a smaller, less expensive system.
2) The use of standard silica optical fibers for transporting optical energy from the laser output to the patient. Pulsed laser systems (required for CR) are plagued by pulse-deteriorating nonlinear effects occurring during their transport through a dispersive media (such as silica optical fiber).^67^ Therefore, pulsed laser systems are often equipped with expensive, specialty optical fibers designed to decrease nonlinear effects. This greatly increases the cost and fragility of a system.
3) The linear nature of SR signal generation means that the light does not need to be focused to produce the SR spectra. Resolution aside, this is an advantage as a larger excitation spot-size decreases the overall irradiance on the tissue.
4) SR is linearly proportional to the concentration of molecules, allowing for direct molecular quantification. SRS shares this advantage. However, there are techniques to achieve linear proportionality with CARS.^48^^,^^68^

CR is best exploited in imaging systems due to its intrinsic optical sectioning and rapid contrast at a single molecular vibrational mode. However, there may be niche uses for CR spectroscopy independent of the imaging capability. Due to the small excitation volume (<10  μm diameter), CR spectroscopy can provide high-resolution sensing, allowing for the delineation of small tissue structures, such as deep brain nuclei. Furthermore, due to the optically sectioned signal, probes can be designed so that excitation occurs far from the fiber tip, even on the other side of protective materials, without sacrificing collection efficiency (as would be the case for confocal SR). This is especially useful if the probe must be placed within a biocompatible sleeve having its own Raman signal at the interrogation wavelength.^69^^,^^70^ Although the small excitation volume also means that the optical energy must be focused and confined to a small volume (therefore limiting the translational value), improved fiber lasers may decrease the required irradiance dramatically.

### 4.3 Rapid Spectroscopic Blood Vessel Detection: An Unmet Clinical Need

There is a clinical risk of hemorrhage when performing closed neurosurgical procedures (i.e., DBS and biopsy) since the surgeon cannot see oncoming blood vessels. Although other optical systems have been presented to fill the clinical need, Raman technologies have not yet been fully exploited for this task. Recently, a translational success was presented using intraoperative OCT for accurate blood vessel detection and size estimation from within a standard biopsy needle during neurosurgery.^18^ Other optical technologies that have investigated blood vessel detection in neurosurgery are LDF and DRS; however, the ability to measure blood vessel size using OCT is a significant advantage for risk assessment.^12^^,^^71^^,^^72^

To minimize the number of optical probes used during a single procedure, it would be ideal to have a probe capable of both blood vessel detection during needle descent and tumor margin detection. Such a probe could be either Raman or OCT, or size permitting, a multimodal combination.

### 4.4 Rapid and Portable Raman Microscopes for Operating Room Histopathology

Histology is time-consuming, requiring fixation, sectioning, and staining of freshly excised tissue. Furthermore, stains require interpretation that can prove challenging or ambiguous even to trained pathologists. Raman technologies, however, provide molecular information with minimal tissue preparation. This would be particularly beneficial in cases where delayed diagnosis could lead to possible repeat surgery because of residual cancer tissue. In this section, we will overview the work that has been done toward vibrational imaging systems for intraoperative *ex vivo* neuropathology, typically with the objective of providing rapid point-of-care pathology information during surgical interventions.

SR microscopes have proven fundamental to dissecting Raman differences in neuropathology;^73^^–^^76^ however, they are traditionally too slow to be used intraoperatively, requiring hours to provide images of tissue slides at microscopic resolution. On the other hand, CR systems built upon the molecular knowledge acquired using SR have shown potential for clinical translation.

The first use of rapid CARS imaging for healthy and cancerous mouse brain tissue delineation was by Evans et al. in 2007.^77^ This was implemented *ex vivo* on an orthotopic human astrocytoma mouse model, and the tumor boundaries were defined by the reduced ${\mathrm{CH}}_{2}$ signal in the tumor regions. Since then, there has been a considerable push for CARS-based histology. In 2014, Uckermann et al.^78^ demonstrated a reduction in lipid signal in infiltrative tumor regions in an orthotopic glioblastoma and brain metastasis mouse model using CARS. By combining CARS with modalities such as two-photon excited fluorescence (TPEF) and second harmonic generation (SHG), detailed images of tissue with structures such as extracellular matrix, blood vessels, and cell bodies could be created. Other groups have further demonstrated the ability of intrinsic TPEF and SHG to aid CARS in brain cancer histology.^79^ Galli et al.^80^ also performed multimodal imaging on excised human tissue samples after 5-aminolaevulinic acid (5-ALA) was preoperatively administered and showed that it did not interfere with the CARS signal. In 2019, the group used the same multimodal approach in an endoscopic setup and demonstrated that the findings were comparable to those *in situ*.^81^

**Fig. 2:**
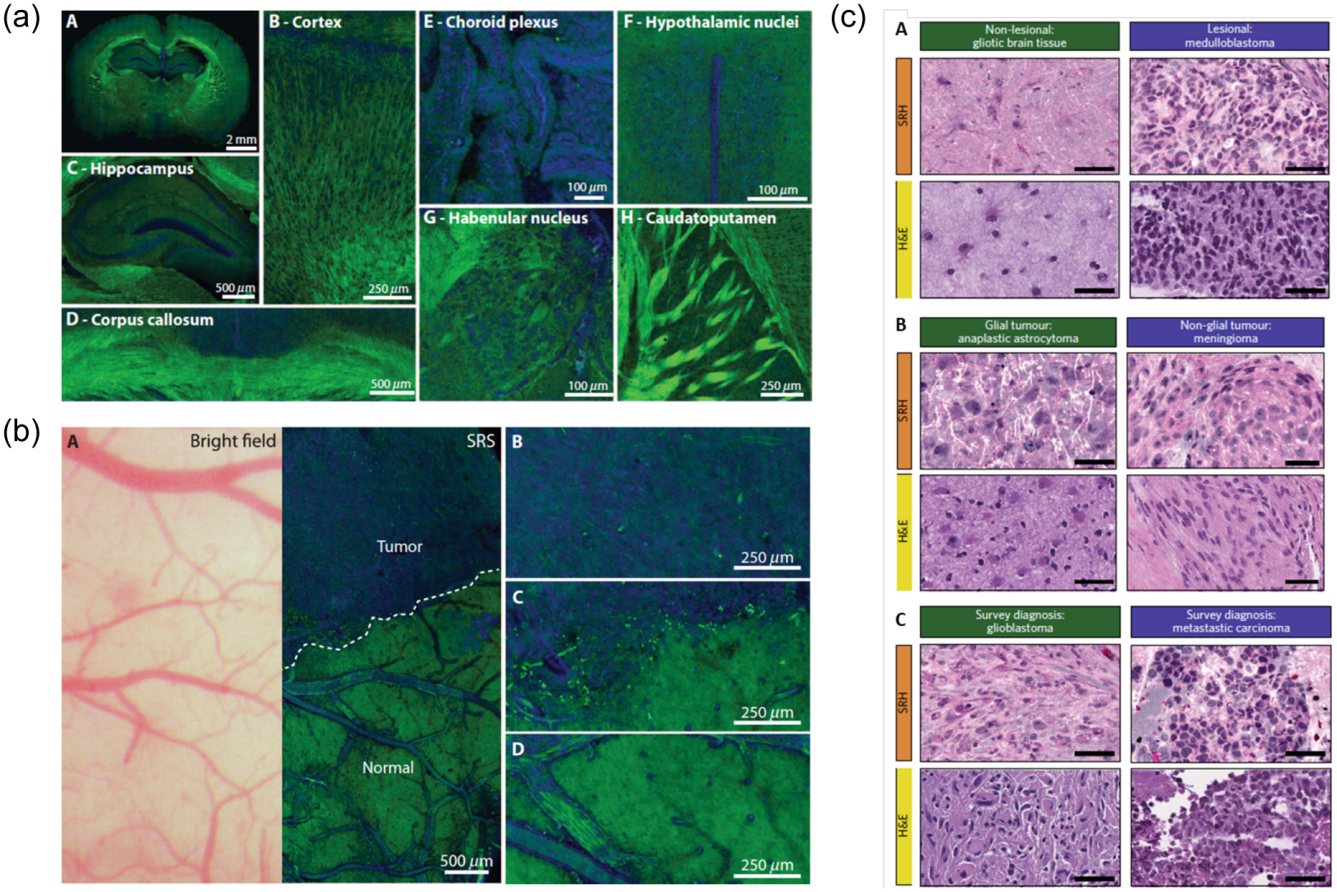
SRS contrast in brain tissue: (a) Epi-SRS images of fresh brain slices from normal mice brain in various brain regions. Lipids are shown in green and proteins in blue. Referring to the inset subfigure labeling: (A) 2-mm coronal slice, (B) cortex, (C) hippocampus, (D) corpus callosum, (E) choroid plexus, (F) hypothalamic nuclei, (G) habenular nucleus, and (H) caudato-putamen.^52^ (b) Brightfield and SRS imaging through cranial window, 24 days after implantation of human GBM xenografts, for comparison of information. Referring to the inset subfigure labeling: (A) Same FOV bright-field and SRS image of xenograft boundary. Brightfield appears normal, whereas SRS microscopy within the same FOV demonstrates distinctions between normal and tumor-infiltrated areas. (B)–(D) Higher-magnification views of tumor (B), at the tumor–brain interface (C), and within normal brain (D). Taken from Ref. 52. Reprinted by permission from AAAS. (c) Comparative examples of processed SRH and H\&E images of gliotic brain tissue, medulloblastoma, anaplastic astrocytoma, meningioma, glioblastoma, and metastatic carcinoma. These images were used in a web-based survey to compare diagnostic outcomes using the two histological methods. Taken from Ref. 54. Reprinted by permission from Springer: Nature.

The main issue with using CARS for histology applications is the low vibrational contrast from proteins, usually represented by ${\mathrm{CH}}_{3}$ contrast. This somewhat limits the ability of CARS images to be directly compared with the gold standard of hematoxylin and eosin (H&E) staining for pathology.^82^ SRS, on the other hand, does not suffer from this shortcoming. In 2012, Freudiger et al.^82^^,^^83^ first used SRS to create images with H&E type information using the vibrational contrast from only ${\mathrm{CH}}_{2}$ and ${\mathrm{CH}}_{3}$ bonds. A year later, the group showed a high correlation between SRS histology and H&E staining (κ=0.98) for glioma detection in mouse brain tissue.^52^ Using a backward-illumination and detection SRS microscope, the group guided the resection of a mouse brain tumor *in vivo* (Fig. 2).^52^^,^^84^ In 2015, the group reported continued progress in using SRS histology to accurately detect and automatically classify tumor infiltrated tissue sections with high accuracy in human brain.^53^ This suggested the feasibility of pathologist-free interpretation of tumor margins for rapid-feedback in the operating room.

**Fig. 3:**
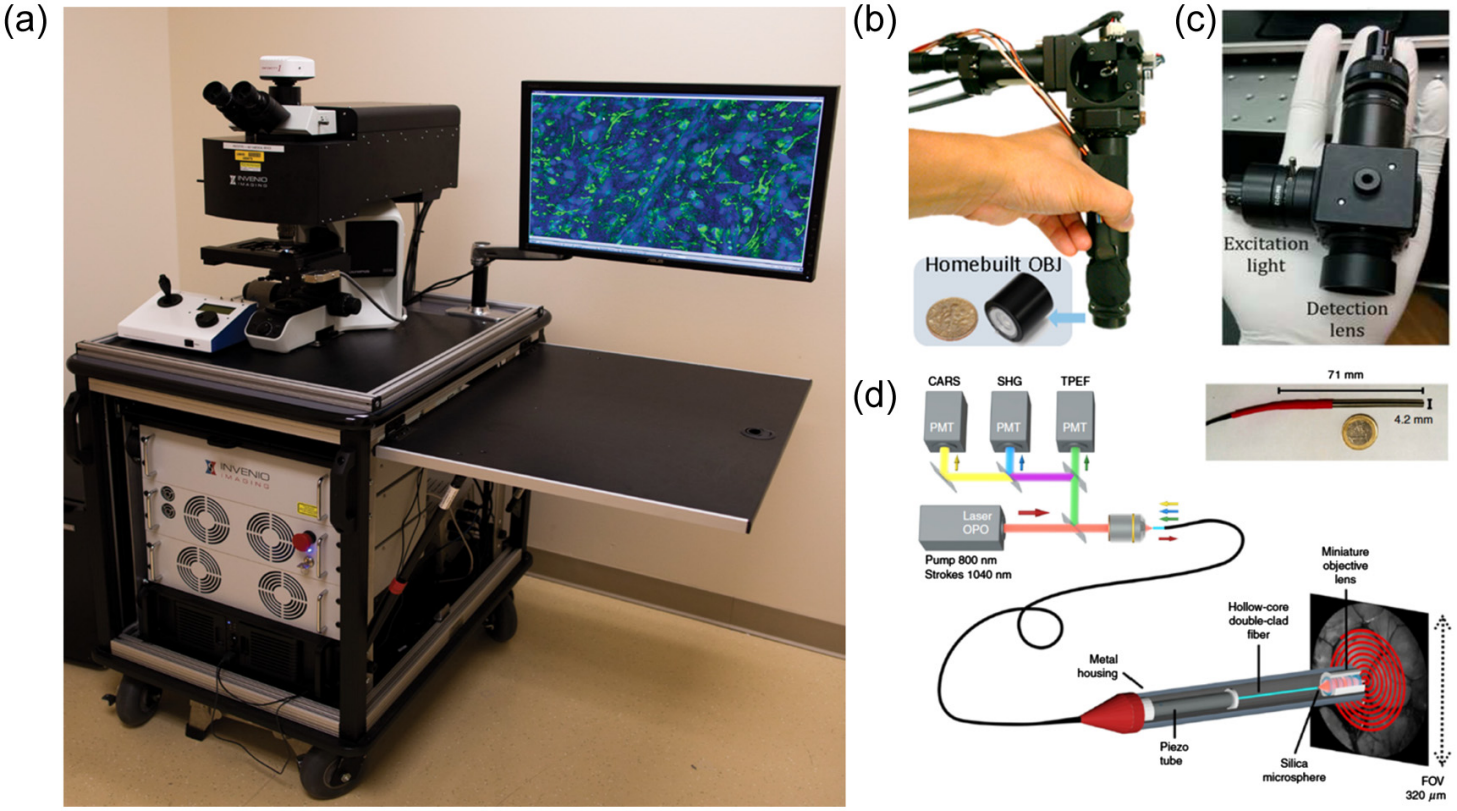
Translational Raman imaging systems. (a) On cart SRS microscope for intraoperative imaging of freshly resected brain tissue. Taken from Ref. 54. Reprinted by permission from Springer: Nature. (b) Fiber-delivered, Handheld SRS microscope. Taken from Ref. 88. Reprinted by permission from American Chemical Society. (c) Handheld widefield SR imager with large FOV. Taken from Ref. 89. Reprinted by permission from Wiley. (d) Multimodal CARS, TPEF, SHG endoscope used to image human colon. Taken from Ref. 90. Reprinted by permission from Springer: Nature.

In 2016, Lu et al.^85^ took the translatability a step further by analyzing fresh human samples, showing that some additional discerning features seen on SRS images were lost in the tissue preparation phase of H&E staining. A particularly innovative aspect of this work is that the images are freely available to help improve diagnostic training in the future.^86^ Made possible by advances in portable and robust fiber laser systems,^87^ the first true fruition of CR *in situ* potential was presented by Orringer et al.^54^ in 2017, wherein the group reported a portable clinical SRS system for intraoperative *ex vivo* neuropathology [Fig. 3(a)]. Using this system, freshly resected tissue sections were compressed and imaged within the operating room. By taking several small field of view (FOV) images, they created interpretable SRS histology mosaics on the order of 2.5 min. In terms of output, the Raman information was used to digitally recreate H&E type staining [Fig. 2(c)] and to perform automatic tissue classification using these images. The optical characteristics of the system are presented in Tables 3 and 4. Furthermore, the system has remained in use for over a year within the operating room without problems or realignment, which speaks to its robustness. This is a point not often mentioned in optical reports but imperative in clinical designs. In 2018, the group reported the use of this system for *in situ* pediatric brain tumor classification with 100% accuracy.^95^

**Table 3:** Optical characteristics of state-of-the-art imaging systems for neurosurgery.

Purpose	Reference	Excitation wavelengths [pump, stokes] (nm)	Power sample surface (mW)	Rep rate (MHz)	Pulse width (ps)	Pulse energy single pulse (nJ)	Peak power single pulse (W)	Peak irradiance single point ($W/{\mathrm{cm}}^{2}$)	Pixel dwell time (μs)	Irradiance image area ($W/{\mathrm{cm}}^{2}$)	Radiant exposure image area ($J/{\mathrm{cm}}^{2}$)
Rapid histology	2017^54^^,^^87^	[790, 1010–1040]	[120, 140]	40	2	6.5	3250	$2.62\times 10^{11}$	2	163	327
	2018^91^	[680–1300, 1041]	[20, 40]	80	2	0.75	375	$3.0\times 10^{10}$	2.4	234	6900
	2019^81^^,^^92^	[781, 1005]	[50]^a^	80^a^	1.2	0.625	520	$7.3\times 10^{10}$	0.4	220	4.5
Handheld imager	2018^88^	680–1300, 1041]	[40, 40]	80	2	1	500	$9.9\times 10^{9}$	6	283	850
	2018^89^	785	500	N/A	N/A	N/A	N/A	N/A	N/A	3.5	300
Endoscope	2017^93^	[816, 1064]	[40, 40]	80	6	1	167	$5.7\times 10^{7}$	N/A	88.9	890
	2018^90^	[800, 1040]	[20,10]	80	0.1	0.375	3750	$1.0\times 10^{15}$	N/A	29.3	23.4
	2018^94^	[817, 1064]	[60, 35]	76	6	1.25	208	$6.9\times 10^{19}$	6	989	376

a Note and Abbreviations: N/A, not applicable or not available. Hard to distinguish from publication, estimates are used. Peak irradiance and single pixel radiant exposure are calculated from the pulse peak power and the airy disk spot size, using the N.A. and wavelength of the published system (Table 4) to derive the spot size from the Airy disk formula ($r_{\text{airy}}=\frac{1.22\times \lambda }{2\times NA_{\mathrm{obj}}}$). Image area irradiance and radiant exposure use the same equations as in Table 1. For the radiant exposure calculation, total image acquisition time is used from Table 4. The wavelength used for all calculations was the one with the highest average power. For SRS this is the higher wavelength, and for CARS this is the lower wavelength. It is critical to note that these values are extremely variable with respect to the optical arrangement; however, they can be useful to consider for clinical systems that aim to image intact tissue.

**Table 4:** Physical characteristics of state-of-the-art imaging systems for neurosurgery.

Purpose	Year	Specimen	Modality	Portability	Orientation	Reported spatial res. [lateral, axial] (μm)	Objective NA	FOV (μm)	Wavenumber acq. time (s)	Spectral range (${\mathrm{cm}}^{-1}$)	Spectral res. (${\mathrm{cm}}^{-1}$)	Total acq. time (s)
Rapid histology	2017^54^^,^^87^	Human fresh resection	SRS	Fibered laser on cart	Transmission	[0.36, 1.8]	1	400×400	2	2845, 2930	N/A	4
	2018^91^	N/A^a^ snap frozen	SRS	Free space optic table	Backward	[0.45^a^, N/A]	1	160×160	0.95	2800 to 3000	15	3
	2019^81^^,^^92^	Human fresh	CARS, TPEF, SHG	Free space optic table	Backward	[0.5, N/A]	1	150×150	0.02	2845	N/A	0.02
Handheld imager	2018^88^	Canine snap frozen	SRS	Handheld optic table	Backward	[1.4, N/A]	0.5	168×168	0.125	2800 to 3000	15	3
	2018^89^	Calf fresh	SR	Handheld (2 cm WD) on cart	Backward	[100, N/A]	N/A	4000×3500	0.7	940 to 1800	120	84
Endoscope	2017^93^	Skin	CARS, TPEF, SHG	8-mm OD endoscope optic table	Backward	[10, N/A]	0.5	$\pi \times 150^{2}$	10	2845	N/A	10
	2018^90^	Colon	CARS, SHG	4-mm OD endoscope optic table	Backward	[0.8, 5.9]	0.45	320×320	0.8	2885	N/A	0.8
	2018^94^	Human nerve cryosection	CARS, TPEF	2.2-mm OD endoscope optic table	Backward	[1.4, N/A]	0.5	310×310	3.8	2841	N/A	3.8

a Note and Abbreviations: Hard to distinguish from article, WD, working distance; OD; outer diameter; and N/A, not applicable or not available. Total image acquisition time was calculated using single-band imaging time multiplied by the number of bands imaged.

Finally, in 2018, Bae et al. presented an epi-illumination and detection hyperspectral SRS system for the subtyping of glioblastomas using HWN spectra.^91^ Technical information about the systems mentioned in this section can be found in Tables 3 and 4.

### 4.5 Toward Raman Endoscopes for Label-Free Imaging of Intact Tissue in Neurosurgery

SR systems have dominated clinical implementation of fiber-delivered spectroscopy, whereas CR systems are the more popular option for preclinical biological imaging applications. This creates a crossroads at imaging endoscopy for the two types of modalities. On the one hand, SR systems used for imaging (i.e., moving beyond single-point) are currently too slow for *in vivo* imaging as the signal is intrinsically weak and dispersed spectrally. On the other hand, CR systems traditionally require bulky lasers and complicated optical transport methods for the high-peak power pulses and provide restricted spectral information. However, there have been considerable technological advances in the past decade for both system types showing that vibrational endoscopy in neurosurgery is close to a reality.

To date, there are few SR imaging endoscopes reported and none have been deployed for neurosurgical improvement. There have, however, been attempts at handheld systems for mesoscopic Raman imaging. St-Arnaud et al.^89^^,^^96^ presented two iterations of a macroscopic wide-field Raman imaging system with ∼1  cm FOV and <400  μm resolution using a multicore imaging fiber for image transport and a tunable filter in the detection path for temporally encoded Raman spectra [Fig. 3(c)]. Although the system required ∼1  min of integration time and relatively high average optical power, the wide-field illumination kept the irradiance levels low. In the future, specific Raman bands could be selected for imaging to decrease imaging time.

CARS endoscopes have been under investigation since 2006, when Légaré et al.^97^ presented backward imaging of polystyrene beads using a single-mode fiber for both illumination and collection. Subsequently, other studies have improved our understanding of the inherent limitations of traditional silica-based CARS endoscopes.^98^^,^^99^ Due to these limitations, specialty fiber optics for pulse delivery with reduced dispersion and background Raman signal have been investigated.^100^^–^^102^

However, CARS endoscopes with the possibility for clinical translation have only recently been reported. Although none of the systems have been used *in vivo* or in brain tissue, these promising candidates for neurosurgical use will be included here. In 2017, Lukic et al.^93^ presented a multicore imaging fiber system that allowed for multimodal CR imaging with no moving parts at the 8-mm outer diameter probe tip. Using this system, they imaged a skin tissue sample with 300-μm FOV and a 10-s acquisition time. Although the probe is quite large for neurosurgery, considerable downsizing could be possible with micro-optical components. In 2018, Lombardini et al.^90^ reported a high-performance CARS endoscope with <1  μm resolution, capable of producing CARS images of a 310×310  μm FOV in only 0.8 s [Fig. 3(d)].The outer diameter of this probe is 4 mm, and images were presented on fresh colon tissue. This system is also capable of variable FOVs and multimodal imaging. The high performance achieved is mainly due to the sophisticated design, using specialty optical fibers (double clad, Kaggome lattice) and a precision-spliced microlens. Although this may seem fragile for the clinic, it is part of the trade-off for high-performance imaging systems. Finally, also in 2018, Zirak et al. presented a 2.2-mm outer diameter rigid CARS endoscope (187-mm in length) for neurosurgery applications. The endoscope was shown to be capable of high resolution, fast CARS imaging with the smallest outer diameter to date.^94^ The technology is enabled by recent advances in GRIN lens technology, whereby long versions of the image-conserving fibers (previously used for *in vivo* CARS endoscopy in mice^46^) are now capable of being manufactured. However, the system still uses large free-space lasers and an optic table for alignment. Therefore, while it is promising, some engineering is required to make the system fibered and ready for the clinic.

In 2010, Saar et al.^84^ presented the first report of fast epi-detected SRS with acquisition times of ∼100  ms using 50 mW for both the pump and Stokes wavelengths. However, the detection scheme in the report used a 10×10  mm photodiode with a hole drilled in the center, through which the excitation lasers were focused. A year later Saar et al.^103^ presented a scanning-fiber-endoscope version of the system using ∼130  mW total power for excitation and the same detection apparatus; *in vivo* work using the device has not been presented since. In 2018, building on earlier work in delay-line tuning, Liao et al.^88^^,^^104^ presented a handheld hyperspectral SRS microscope capable of HWN spectroscopic images ($15\;{\mathrm{cm}}^{-1}$ spectral resolution) on the order of 3 s [Fig. 3(b)]. Using this system, the team imaged sections of healthy and cancerous canine brain tissue, but did not go into much detail on the ability to distinguish the two samples. Technical information about the systems mentioned in this section can be found in Tables 3 and 4.

### 4.6 CARS or SRS Endoscopy

SRS has improved nuclear contrast in comparison with CARS due to a reduced nonresonant background that allows for faster imaging speeds of molecules at low concentrations.^48^^,^^83^^,^^105^ However, successful systems imaging in the backward (epi) direction using SRS are scarce in comparison. In microscopy, this is likely because CARS can be more easily incorporated into traditional laser scanning microscopes used for TPEF and SHG. In clinical applications, however, the main hurdle for SRS is the increased complexity of signal detection, made even more complicated with the push for portable fibered lasers. Gottschall et al.^106^ provide a good resource for understanding the benefits of each CR modality in their review on advances in laser concepts for multiplex CARS.

Another factor that can play a considerable role in clinical applications is the operability of the system under ambient lighting. SRS has an advantage here for two reasons: (1) the modulated signal can easily be distinguished from background contributions and (2) the wavelength of detection is further into the near-infrared region. Although traditionally considered an advantage of CARS in microscopy, the blue-shifted signal generation is a burden in the clinic as the detected signal is often near the visible region where there are strong surgical lighting contributions. This could be circumvented by pushing CARS sources further into the NIR region.

## 5 Optical Exposure to Brain Tissue

There are limits to the amount of optical irradiance that can be introduced into a biological system without causing serious cellular damage. The maximum permissible exposure (MPE) of continuous-wave (CW) optical radiation for tissues such as the retina and the skin can be calculated from international standards.^107^^,^^108^ There is also an inherent danger of laser light accidentally being shone directly into an eye at any point during a laser’s operation. Although this is a serious challenge in designing clinical laser instruments for ethics approval and eye-safety, here we speak more about the dangers of deliberate laser–tissue interactions and assume that the proper safety eye-wear, or other appropriate risk mitigation strategies (e.g., laser activation only upon tissue contact with an imaging probe), are being used in the operating room.

Although the international standards provide a convenient calculation for the MPE of CW radiation, they should not necessarily be used as a guideline for Raman systems investigating brain tissue. The optical and thermal properties of skin and retina differ considerably from brain tissue, and the higher water content of the brain results in a lower conversion of photon energy to thermal energy.^109^^–^^111^ Moreover, the standards themselves are not designed for deliberate laser exposure during medical procedures.^107^^,^^108^ To make matters more complicated, brain sensitivity to thermal damage is somewhat unclear. Reports of minor local temperature changes of only 2°C have been shown to cause thermal damage to metabolically active brain cells.^111^ However, it has also been shown that the awake animal brain naturally fluctuates in temperature within this range of 2°C.^112^ Furthermore, in terms of photothermal damage at the cellular level, it has been shown that injuries are reversible for temperatures that have increases of 6°C.^113^

The photo-induced effects of CR systems are particularly complicated due to (1) high-power density, (2) focal point scanning, and (3) nonlinear damage such as photochemical ablation and optical breakdown.^114^^–^^116^ Due to short-pixel dwell times in rapid focal point scanning, instantaneous heating through linear absorption is often considered negligible in comparison with nonlinear damage for multiphoton systems.^114^^,^^115^ However, continuous scanning of the same FOV can produce a volumetric heating effect that must be accounted for, as shown in Refs. 72 and 117. In 2001, Hopt et al. described a general formula for the tissue damage rate D that was proportional to the optical intensity raised to the n’th power ($P^{n}$), the repetition rate ($f_{\text{rep}}$), and the pulse width ($\tau_{\text{pulse}}$) of the laser. Using this information, Hopt and Neher explained an optimal CR system that can maximize signal while minimizing damage.^114^ Such a system for clinical CR would operate in the NIR (pump and stokes=1000 to 1500 nm), have a relatively low repetition rate near 1 MHz, and use pulsewidths between 1 to 10 ps, operating at about 30 mW total average power.^114^

## 6 Data Analysis for Spectroscopic Information

Working with Raman data presents multiple challenges: Raman signal is intrinsically weak, and complicating factors such as excess blood, surgical lighting, and device manipulation can exacerbate this. Furthermore, living tissues are complex and dynamic systems, composed of thousands of interacting molecules that are heavily influenced by external factors.^118^^–^^120^ Competencies at the intersection of signal processing (spectral, image-based, or a combination of both), data mining, and machine learning (ML) are essential to the design of cutting-edge biomedical RS systems.

### 6.1 Spectra Data Processing

**Fig. 4:**
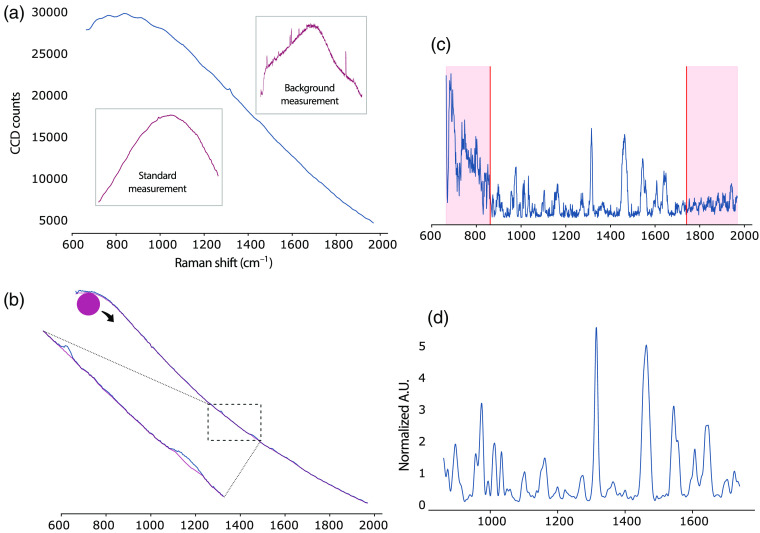
Spectral processing of raw Raman signal in biological tissue. (a) Raw Raman measurement (blue): during the acquisition, a background measurement is also recorded with the laser turned off to correct for ambient light. After each experiment, an acquisition on a Raman Standard with known Raman response is used to correct for artifacts from the acquisition system. (b) Baseline correction: a curve fitting algorithm (pictured here: rolling ball algorithm) is used to estimate the shape of the baseline signal, which mainly consists of tissue autofluorescence. This baseline curve is then subtracted from the acquired signal. (c) Truncation to desired spectral range: spectral regions with poor Raman information or exhibiting artifacts from the experimental design (e.g., silicate substrate) or correction algorithm are removed. (d) Smoothing and normalization: high-frequency noise is removed from the signal and the spectra are expressed in normalized units so that they can be compared across samples and experiments. A.U., arbitrary units.

Raw Raman acquisitions are characterized by low Raman signal, high amounts of shot noise, and intense signal from background sources (e.g., AF and ambient light in SR, coherent background in CARS).^63^ Signal processing aims to maximize the Raman component of the acquired signal while minimizing the contribution of these other processes. As an example, in SR, a complete signal processing pipeline generally includes truncation of the signal to the desired spectral range, correction for ambient light, cosmic ray removal, correction of the spectra for the system response with a standard measurement, background removal, smoothing of the spectra to remove high-frequency signal associated with shot noise, normalization, and data quality assessment (Fig. 4).^119^

Following cosmic ray removal and correction for system response, the most important remaining signal contribution in SR is AF, a spontaneous process resulting from the emission of light from endogenous tissue fluorophores including elastin, tryptophan, and nicotinamide adenine dinucleotide.^121^ Even with proper hardware, AF intensity can be orders of magnitude higher than that of Raman scattering.^122^ AF results in a broad spectrum that can underlie the narrow Raman peaks, making baseline estimation a critical step in the signal processing routine. Many techniques exist to mathematically estimate and remove the background in SR, leveraging the smooth and predictable decay of its contribution throughout the spectral range. Polynomial fitting of the spectra is the most widely used technique, but it heavily relies on expert knowledge to select the proper parameters and avoid over- or under-fitting the signal.^119^^,^^123^ To create completely automated routines, more recent algorithms that rely on iterative fitting of the signal with sophisticated cost functions ensuring an improved fit while minimizing expert intervention have surfaced.^124^^–^^127^ Nevertheless, in biological experiments, the background-generating processes are not always clearly identified, resulting in a correction that is based more on spectral morphology than exact comprehension of the underlying phenomenon. Some authors even argue against this step to avoid altering the spectral shape in unpredictable ways.^128^

### 6.2 Data Analysis Methods

Although RS has historically relied on the visual assessment of the spectra, the complexity of the signal acquired in living tissue has necessitated the reliance on automated algorithms.^129^ Most clinical applications of RS for neurosurgery rely on three types of analytical tasks: supervised ML (classification), spectral imaging, and biomolecular interpretation of spectral features.

#### 6.2.1 Supervised machine learning

**Fig. 5:**
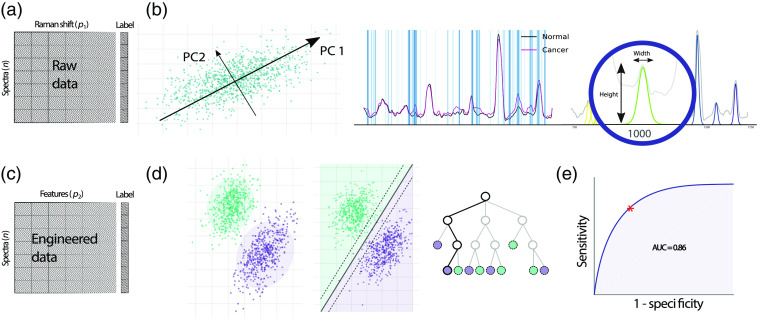
ML for RS. (a) The initial data matrix is composed of n spectra and $p_{1}$ variables, where each variable represents an intensity at a particular wavelength. A standard, processed Raman spectrum can contain between 500 and 1000 variables. Each spectrum is associated with a label (e.g., high-grade glioma versus normal tissue). (b) Feature engineering algorithms. These algorithms are designed to change the representation of the initial spectra matrix into one that will enhance performances of the ML predictions. From left to right: PCA, feature filtering, and feature extraction (peak fitting). Each method is described in the main text. (c) The engineered data matrix. Each spectrum now contains $p_{2}$ variables, which are the results of the previously applied feature engineering methods. These variables could be PC scores, intensity at specific Raman shifts, peak-fitted peak height, etc. (d) Supervised learning algorithms. Different mathematical functions can be trained to separate the spectra with distinct labels based on the values of their variables. From left to right: LDA, SVM, and decision tree. (e) Testing of the ML model. The different ML pipelines (including feature engineering) are trained on a subset of the data and tested on an independent subset. The predictions of the models are compared with the true labels of the testing set, and prediction performance is evaluated. A ROC curve can be drawn to estimate the performance of the prediction at varying levels of sensitivity and specificity. The best performing threshold is the point on the curve closest to the left upper corner (red star). PC, principal component; PCA, principal component analysis; and AUC, area under the curve.

Supervised learning consists of training an algorithm, for example, to recognize tissue phenotype from its spectral signature. A data matrix $X_{n\times p}$ containing n spectra represented each by p variables or features (e.g., the signal intensity value for each wavenumber) is associated with a vector of labels y (e.g., the tissue diagnosis from the neuropathologist) [Fig. 5(a)]. The algorithm or function f for which f(X)=y^ is optimized to minimize the loss function L(y,y^), where y^ is the model-predicted tissue label. The result of the loss function is called the training error. The testing error is calculated from predictions on new data not used for training. Common supervised learning algorithms are described in Table 5.

**Table 5:** Feature engineering and supervised ML algorithms for classification of RS in brain tissue

Task	Technique	Description	Pros	Cons
Feature engineering	PCA	Unsupervised algorithm. Extracts the projection of the data matrix onto a set of linearly uncorrelated principal components.	Agnostic to tissue classes (unbiased)	Expert intervention is impossible
			Effective feature reduction	Agnostic to tissue classes (can reduce classification performances)
			Fast	
			Removes correlated (redundant) features	Susceptible to features unrelated to the classification task (e.g., noise and artifacts)
			Effective visualization technique	
	Feature extraction	Generate new variables from the spectra (e.g., band fitting) or by transformation of already available variables (e.g., peak ratios).	Increases the information content of the data	Requires expert knowledge
			Interactions are accounted for	Time and effort intensive
			Better captures molecular processes	Requires high data quality
	Feature selection	Identification of the variables that show highest correlation with class (filter method). Can be performed as a second feature-engineering step, after PCA or feature extraction.	Simple	Biased
			Task specific	Prone to false positives
Supervised learning	LDA	Estimates a set of multivariate normal distributions that better explain the data and assigns new observations to the class with highest likelihood.	Simple	Assumes normality of distribution
			Fast	Assumes homoscedasticity
			Low data requirements	Requires independent variables
	SVM	Finds a hyperplane that maximizes the margin between the support vectors, i.e., the observations of each class closest to the hyperplane.	Adaptable to nonlinear feature spaces	Performance is highly dependent on hyperparameters
			Fast when N is small	Requires engineered features
			Easily implemented	
	Decision trees	Nonparametric model that assigns a decision function to each variable. Decision trees can be aggregated to increase stability by averaging (boosted trees) or training over many subsamples (random forests).	Intuitive	Unstable by itself
			Able to model complex feature spaces	Aggregation is time consuming
	ANN	Connectionist model that considers each variable an input neuron. These neurons are subsequently connected to a predefined number of hidden layers through an activation function. These hidden layers connect to an output layer that generates the class prediction. ANN with more than one hidden layer are called deep neural networks.	Adapts to any nonlinear function	High data requirements
			Highly customizable	Performance is highly dependent on hyperparameters
			High performances	
			Does not require feature engineering (deep neural networks)	Computationally intensive

Feature engineering is the transformation of the processed signal into a set of variables that will be used as representation for the learning task. It ranges from selecting a subset of all available intensities associated with different wavenumbers to generating new variables and performing complex mathematical transformations of the data to unveil properties not necessarily conveyed by the original spectrum. In RS, this transformation is critical because of two properties of the data: high dimensionality and sparsity of the feature space.^130^ High dimensionality refers to the large number of available features (e.g., between 500 and 1000 spectral bands in SR) needed to describe a single observation. Feature sparsity means that most of this information will be unhelpful in discriminating different tissue phenotypes: all tissues are composed in majority of the same organic molecular compounds and many of the molecular markers are redundant across the spectral range.

In the neurosurgical literature, the most common feature engineering algorithm is principal component analysis (PCA).^56^^,^^59^^,^^74^^,^^76^^,^^128^^,^^131^^–^^139^ PCA iteratively finds the orthogonal vectors (or principal components, PC) that maximize the variance in the dataset and then stores the projection of the data point upon each PC [Fig. 5(b)]. The PCs are ranked by their eigenvalues, or amount of variance explained; therefore, most of the variance in the dataset can be expressed in the first PCs. Subsequent PCs can be discarded, resulting in a compressed dataset. The number of retained PCs ranges between 2 and 40 depending on either predefined criteria such as amount of explained variance^55^^,^^56^^,^^74^^,^^134^^–^^136^^,^^138^^,^^139^ or *post hoc* criteria, e.g., selecting PCs that could better differentiate the different tissue types.^76^^,^^131^^–^^133^ The popularity of PCA in RS can be attributed to its unsupervised nature: as it is agnostic to labels, it is considered unbiased.^140^ Moreover, most authors report that over 99% of the variance of their dataset is expressed in the first 2 to 40 PCs^55^^,^^56^^,^^73^^,^^134^^–^^136^^,^^138^^,^^139^ and the orthogonality of the extracted features can improve the efficiency of classical multivariate linear models.^141^

Another feature engineering technique involves selecting a small subset of the best features and discarding all others. These features can be identified by (1) simultaneously assessing their individual correlation with the outcomes (called filter methods),^142^^–^^145^ (2) iteratively evaluating changes in ML performances when excluding/including each feature into the model (wrapper methods),^146^ or (3) adding penalization terms to the optimization of the training algorithm so that some of the features’ contributions are reduced to zero (embedded methods).^147^ The features are selected either directly from the processed spectra or from previously extracted features, as part of a two-step feature engineering pipeline.^131^^–^^133^^,^^136^^,^^148^ Most studies involving brain tissue rely on filter methods to identify important features,^149^^,^^150^ and in some cases, features are selected manually.^148^^,^^151^^,^^152^ In proteomics and genomics studies, performance-based and optimization-embedded have proven superior to filter methods in recovering truly important features in sparse, high-dimensional datasets.^153^^,^^154^ Embedded methods have recently started to emerge in the biomedical Raman literature to identify crucial features, but not as part of a supervised learning task.^155^

The last feature engineering strategy is the generation of new features from the existing data. For example, the height ratio of two peaks can carry important information such as summarizing the lipid-to-protein content of a sample. Peak ratios have proven useful in discriminating white and gray matter in the brain and in differentiating between normal, necrotic, and malignant tissue. The shape of the Raman spectrum can also be used as marker of malignancy.^148^^,^^155^ In their study, Stables et al. selected eight target bands, for each of which they calculated the centroid (weighted mean of the signal in the defined region), skew (asymmetry of the intensity values), and kurtosis (prominence of certain intensity values from the rest of the bands). From this, a sequence of 10 to 30 contiguous variables within a Raman tissue band can be represented by two or three features or parameters, resulting in enhanced ML performances [Fig. 5(b)]. Although this approach to feature extraction is extensively used in the Raman literature,^156^ it is rarely use in neurosurgery-related research.^140^^,^^157^ Opponents of band fitting for biological Raman signals argue that the selection of target bands cannot be reliable in the case of low SNR signal exhibiting a high number of potential peaks and that our knowledge of Raman generating processes in tissue is not strong enough to limit our analysis to a few critical bands.^140^^,^^157^ Over time, more authors may begin to incorporate band-fitting routines to the analytical pipeline as it is an effective way to summarize the vibrational profile of a tissue in a biochemically meaningful manner, while allowing for easy statistical manipulation and even further feature engineering.^155^

#### 6.2.2 Classification

The classification tasks comprise of the selection, training, and evaluation of an ML model that will map the engineered feature matrix to a vector of observed tissue classes [Figs. 5(d) and 5(e)]. Linear discriminant analysis (LDA) is the most tried and tested Raman classification model in RS. LDA assumes that all observations with the same label originate from multivariate normal distributions with equal covariance and assigns new observations to the label with highest likelihood. Because it is vulnerable to highly correlated features, LDA is often preceded by PCA. Despite its widespread adoption, LDA relies on a set of assumptions that are not respected by Raman data: multivariate normality of the distributions, independence of the predictors, homoscedasticity (homogeneity of covariance across labels), and few outliers.^158^ Furthermore, because of the often inconsistent SNR characteristics of biological acquisitions, researchers need algorithms that are robust, i.e., that are not overly sensitive to noise in the data. The complexity of biological Raman data and easy access to ML libraries have motivated the reliance on more flexible models for this problem.

The support vector machine (SVM) learning model finds the hyperplane that optimizes the distance between itself and the closest point of each class it tries to separate.^159^ Using kernels, SVM can adapt to a nonlinear feature space with minimal computational cost. Authors that have used SVM were able to separate up to seven tissue classes with acceptable accuracy using SR signal from *ex vivo* brain tissue.^134^^,^^138^^,^^148^^,^^160^ However, extensive feature engineering was necessary in all cases. In the anticipation of the translation to clinical practice, other types of models are being explored to further increase predictive performances of Raman-based systems. Decision trees are nonparametric models in which each internal node represents a decision function based on an input variable. Ensemble methods such as bagging and boosting, which work by aggregating multiple decision trees trained on different subsets of the data, can significantly improve the performances of decision trees.^161^ Boosted decision trees have shown success in classifying between normal tissue and glioma without using any prior feature engineering.^11^^,^^57^ Artificial neural networks (ANN) have received the most attention in ML research over the last few years due to their potential deep structure and their ability to adapt to virtually any possible function.^162^ In neurosurgical applications, Jermyn et al.^64^ showed how a simple ANN could reduce interference with surgical ambient light, while performance of other models was strongly affected in such conditions. Deep neural network architectures such as convolutional neural networks (CNN) have shown great promises in biospectroscopy, with the additional benefit of being less dependent on spectral preprocessing.^163^^–^^169^ Data requirements to train and optimize such models are high because of the millions of parameters they contain;^170^^–^^172^ nevertheless, open access to large Raman datasets and strategies such as transfer learning and novel data augmentation methods (such as the simulation of Raman spectra for DRS analysis^173^) will make their adoption possible for biomedical applications in the near future.^174^

#### 6.2.3 Single band to hyperspectral imaging

Raman measurements acquired at regular intervals over a sample can be assembled into a Raman image in which each pixel contains a Raman spectrum. Raman imaging can solve several limitations of point probe systems as they can better resolve the heterogeneity of a sample and can work across scales, from microscopic imaging to widefield, macroscopic imaging. Furthermore, the contrast in these hyperspectral images can be tuned to contain varying levels of molecular significance, depending on the way the spectra are processed and analyzed.

**Fig. 6:**
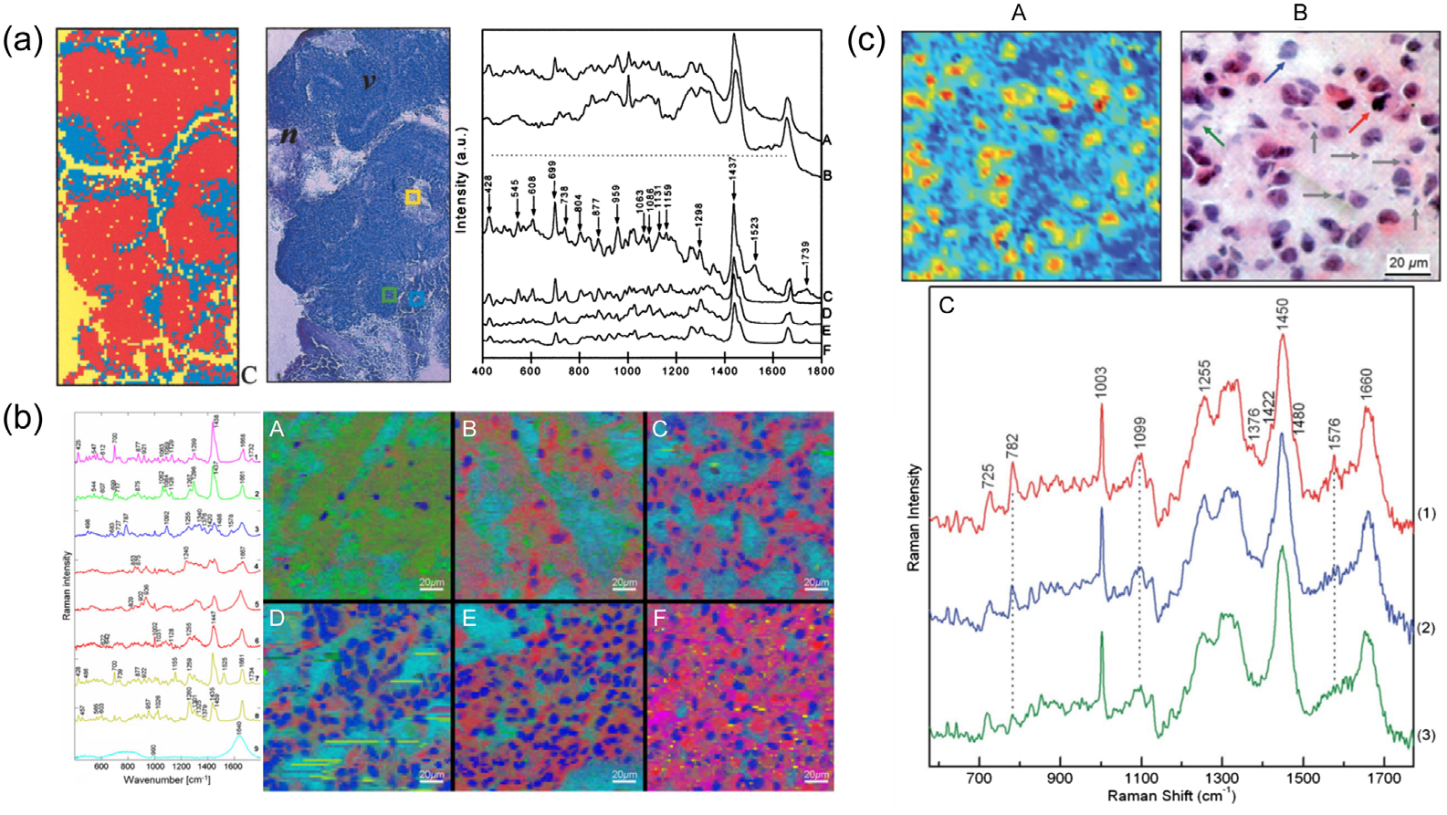
Spectral unmixing and clustering-based approaches to Raman imaging in which every pixel consists of an entire processed Raman spectrum. (a) ML-based approach. A supervised ML model was used to assign each spectrum acquired in glioblastoma tissue to one of two classes: vital tissue and necrotic. Left: the Raman image, color-coded with the predicted class for each pixel (red: vital, blue: necrotic, and yellow: background). Center: H&E image of the sample (v: vital and n: necrotic). Right: The averaged spectra of the (A) necrotic and (B) vital samples, with the (C) difference spectrum compared with (D) cholesterol, (E) cholesterol oleate, and (F) cholesterol linoleate. Taken from Ref. 136. Reproduced by permission from Springer: Nature. (b) Endmember-based approach. The N-FINDR unmixing algorithm was used to identify a prespecified number of endmember spectra from a dataset acquired in glioma tissue. Each spectrum in the dataset is then expressed as a linear combination of these endmembers. The endmembers were assigned to cholesterol ester (1, magenta), phopsholipids (2, green), DNA (3, blue), proteins (4 to 6, red), beta-carotene (7, yellow), unsatturated fatty acids (8, yellow), and phophate buffer solution (9, cyan). Each pixel in the Raman image is then colored based on the relative abundance of each endmember in its spectrum. Taken from Ref. 74. Reproduced by permission from Springer: Nature. (c) Endmember-based approach. VCA was used to find three endmember spectra in a dataset acquired in glioblastoma samples. Each endmember was assigned to an RGB channel (spectra 1, 2, and 3). The relative abundance of each endmember in every pixel is used to color-code the Raman image (A). The H&E image (B) is provided as a comparison. Taken from Ref. 128. Reproduced by permission from the Royal Society of Chemistry. VCA: vertex component analysis.

Unsupervised learning is a family of ML algorithms that does not rely on the prelabeling of each observation; instead, they serve to unveil hidden patterns in the data. An example of unsupervised learning is clustering: the dataset is divided into several groups or clusters in which the similarities between spectra in a cluster are maximized compared with the dissimilarities with spectra from other clusters. This method has the advantage of accounting for the entire spectral information available and not just one or two defined bands. Early adopters of this technique were Koljenovic et al.,^136^ who used PCA and clustering to assign each pixel to one of 70 to 72 distinct clusters. A supervised model was then trained to map a cluster to either necrotic or vital tissue, on which the final contrast of the images was based. Other authors have used clustering, with or without prior engineering, to assign each pixel to a specific cluster and generate the contrast^135^^,^^175^^,^^176^ [Fig. 6(a)].

When more than two clusters (or colors) are used, clustering-based images can become challenging to interpret. Recent approaches attempt to overcome this limitation and create images more amenable to visual interpretation. As SR signal is a linear combination of the individual Raman signals of every molecular compound in a tissue, recovering the coefficients of this linear combination can uncover the relative quantity of important molecules from the tissue Raman spectra. This approach has been referred to as hyperspectral unmixing. The different methods for linear unmixing are based on three elements: the selection of the basic, reference substances or endmembers, their spectral signature, and their relative abundance. In brain tissue, vertex component analysis (VCA) and N-FINDR are two such algorithms. Both are unsupervised and differ mostly in their definition of endmember spectra. In N-FINDR, reference spectra from pure molecular substances are set by the user, and endmembers are set as the spectra with highest correlation to those reference substances. In VCA, the endmembers spectral signatures are mathematically identified based on the variations in the dataset. Importantly, both methods assume that signal from pure substances are present in the data and that the molecular compounds are independent.^177^^,^^178^ Applied to brain tissue, VCA and NFINDR were used to create pseudocolor images with RGB channels, with each channel representing the relative abundance of one of three endmembers^128^ [Fig. 5(c)]. The number of distinct channels used to create images varied from 3 to 9 in some studies, with higher numbers yielding images able to discriminate between more different molecular components^73^^,^^74^ [Fig. 6(b)]. With this approach, researchers were able to generate metrics such as nucleic acid content, lipid content, and lipid-to-protein ratio, which correlated with malignancy in astrocytoma samples.^74^ An issue with the spectral unmixing of Raman pixels is the difficulty in assessing the reliability and variance in both the endmembers definition and the relative abundance estimation. Although spectral unmixing has the advantage of harnessing information from the entirety of the Raman spectral range, more recent work settled for extraction of specific, predefined spectral features such as band ratios and single-band intensities to represent independent molecular information.^75^^,^^179^ Nonetheless, it demonstrated a 90% pixel-to-pixel classification accuracy between white matter, gray matter, and pathological brain tissue (glioblastoma, necrosis, or infiltrating cancer).^75^

Raman images can help pathologists visualize molecular information not present in H&E stained samples, but they could also be amenable to ML classification algorithms. This would considerably reduce time-to-diagnosis during neurosurgeries, where the aggressiveness of the resection can be dictated by a preliminary diagnosis provided by a neuropathologist from a frozen tissue sample. Recently, Hollon et al.^180^ used a CNN to classify Raman-based images of freshly excised brain tissue into one of 13 histologic categories. In a prospective, multicenter study, they demonstrated a classification accuracy of 94.6%, whereas automated classification based on conventional H&E staining was 93.9%.

#### 6.2.4 Biomolecular identification of spectral features

Despite inferring a diagnosis for a tissue sample, Raman spectra can be interrogated to describe the biochemical content of a sample. In a static system composed of a single molecular compound acquired in perfect experimental conditions, the Raman features of the distinct vibrational modes are directly observable, and subtleties such as a slight peak shift or peak widening can be used to derive changes in the system.^181^ In biological tissue, computational strategies are needed to deconstruct the more complex Raman spectra.^163^^,^^182^

**Fig. 7:**
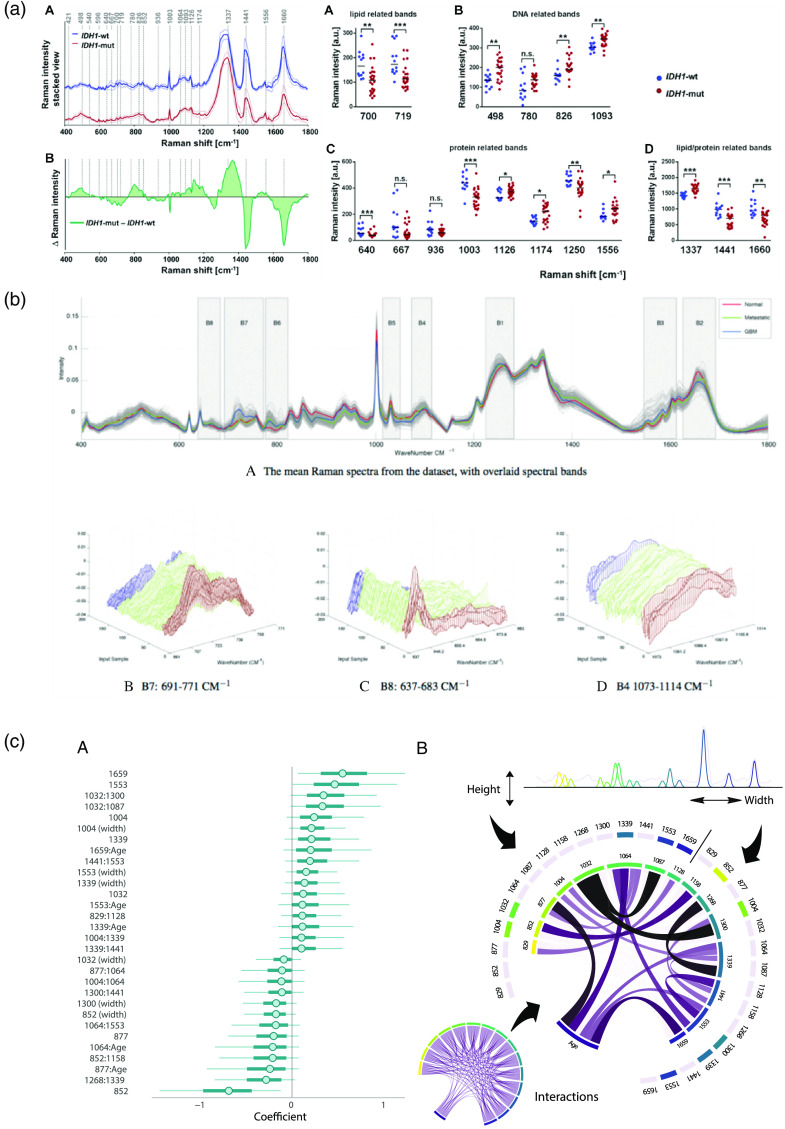
Chemometrics analysis of Raman data. (a) Comparison of IDH mutation status in samples of human glioma. (A), (B) the difference spectrum (green) between IDH-wt (blue) and IDH-mut (red) averaged spectrum is used to identify important Raman markers. (A)–(D) Univariate analysis of the distribution of Raman intensity values at different Raman shifts. Taken from Ref. 151. Reproduced by permission from Springer: Nature. (b) Spectral shape analysis of normal brain, glioma, and metastatic human samples. The target regions are selected (a). For each region, parameters describing the shape of the distribution of Raman intensities inside the regions are extracted and compared between the three classes (red: normal, green: metastatic, and blue: glioma). Taken from Ref. 148. Reproduced by permission from the Royal Society of Chemistry. (c) Analysis of importance of spectral markers between high-density and low-density/normal brain samples from *in vivo* human brain tissue.^155^ (A) The coefficients of a multivariate linear model are plotted. Features with a positive coefficient value are more prominent in samples with high cancer density, while negative values are associated with low or absent cancer density. (B) Visual representation of the spectral markers (peak height, peak width, peak-to-peak and peak-to-patient-age interactions).

A widespread approach is the comparison of specific spectral features (e.g., peak heights, band intensities, and band-to-band ratios) between the averaged spectra of each tissue phenotypes [Fig. 7(a)]. These bands are recovered either from the difference spectra between two tissue phenotypes or by analyzing the relative importance of each Raman bands after applying PCA. In brain tissue, Raman signal originates predominantly from nucleic acids (782, 829, and $1339\;{\mathrm{cm}}^{-1}$), lipids (1063, 1086, 1131, 1268, 1300, 1441, 1659, 1670, and $1739\;{\mathrm{cm}}^{-1}$), amide I ($1659\;{\mathrm{cm}}^{-1}$) and III (1268 and $1300\;{\mathrm{cm}}^{-1}$), and amino acids such as tyrosine and proline (829, 852, and $877\;{\mathrm{cm}}^{-1}$), tryptophan ($877\;{\mathrm{cm}}^{-1}$), and phenylalanine (1004 and $1032\;{\mathrm{cm}}^{-1}$). For the HWN region, the CH-stretching of lipids (2845 and $2885\;{\mathrm{cm}}^{-1}$) and proteins ($2930\;{\mathrm{cm}}^{-1}$) are the predominant molecular markers. As a result of its high cellularity and myelin content, lipids are the brain tissue’s main constituents.^175^^,^^183^ The ${\mathrm{CH}}_{2}─{\mathrm{CH}}_{3}$ deformation at $1441\;{\mathrm{cm}}^{-1}$ often dominates the spectrum. The signal from amide bands is also important, but it strongly overlaps with lipid bands.^56^^,^^131^^,^^184^ Markers of glioma include the phenylalanine band at $1004\;{\mathrm{cm}}^{-1}$, the nucleic acids/${\mathrm{CH}}_{2}─{\mathrm{CH}}_{3}$/amide III band at $1339\;{\mathrm{cm}}^{-1}$, and the carotenoid bands at 1159 and $1523\;{\mathrm{cm}}^{-1}$.^56^^,^^75^^,^^131^^,^^136^^,^^149^^,^^152^^,^^183^^–^^185^ For other bands, studies show conflicting associations. In some cases, the nucleic acid signal is strongest in tumor and necrotic tissue, whereas others demonstrate a decrease in malignant regions. The disagreements on how Raman signal changes as a result of pathological states stem from the multiple differences in experimental design from one study to another (e.g., which types of tissue are compared, spatial resolution of the systems, tissue processing before Raman acquisition, and analytical methods used to process and compare the Raman signals), along with low sample sizes (often <10  patients).^186^

Peak ratios are also considered to effectively summarize the lipid-to-protein content of a sample, with good discriminating power between white and gray matter and between normal brain and cancer. In these cases, involved bands included the $1442:1662\;{\mathrm{cm}}^{-1}$ (or $1441:1659\;{\mathrm{cm}}^{-1}$) peaks,^75^^,^^187^^,^^188^ the $1299:1439\;{\mathrm{cm}}^{-1}$ peaks,^56^ the $1266:1300\;{\mathrm{cm}}^{-1}$ peaks,^189^ and the $2930:2845\;{\mathrm{cm}}^{-1}$ peaks.^19^^,^^52^ Although simple and easily interpretable, these methods of spectral interpretation are limited by their inability to model complex interactions between the molecular species present in the living tissues.

As presented in the previous section, Raman linear unmixing (estimating the relative contribution of different pure endmembers to the total Raman signature) can help recover the tissue’s biological content. Using VCA or NFINDR algorithms, authors concluded that cholesterol esters, nucleic acids, collagen, and carotene contributions were higher in high-grade tumor while general lipid content and both lipid-to-protein and lipid-to-DNA ratios were decreased in malignant tissue.^73^^–^^75^^,^^179^ However, the reliability and consistency of this approach in unveiling the relative quantity of the molecular compound in the interrogated sample has not been thoroughly validated. It does rely on two important assumptions that may not hold true in biomedical applications: (1) the dataset of Raman spectra contains signal from pure molecular endmembers and (2) the quantity of each molecular endmember is independent of the others.^177^^,^^178^

In reaction to this limitation, new approaches to the interpretation of biological Raman data that embrace the complex structure of the data-generating process and integrate into their models the uncertainties around the Raman signal and sample diagnosis have emerged. Stables et al.^148^ selected spectral regions with high discriminating yield between glioma and normal brain samples, for which they extracted markers of Raman intensity distribution [Fig. 7(b)]. These features carry more biochemical significance and stability than a signal at a single Raman shift. The authors also calculated the ratio between every pair of extracted features as an additional feature ensemble, expanding the idea of interactivity between spectral information to all available variables. Their results demonstrated that the 691- to $771\text{-}{\mathrm{cm}}^{-1}$ spectral region associated with phospholipids and amino acids had the highest discriminatory power, followed closely by 637- to $683\text{-}{\mathrm{cm}}^{-1}$ (nucleic acids and amino acids) and 1073 to $1114\;{\mathrm{cm}}^{-1}$ (nucleic acids and phospholipids). However, the samples were formalin fixed paraffin processed and Raman signal was altered to remove the paraffin-associated bands, which could explain some discrepancies between their findings and findings from fresh or frozen tissue. Nevertheless, this work was the first to incorporate systematic interactions between spectral feature as a potential discriminator between tissue classes, a key step in bridging the gap between classical Raman analysis and more complex computational pipelines used in other data-driven technologies such as neuroscience, genomics, and proteomics.

In Ref. 155, a feature engineering process integrating domain-specific knowledge, band-fitting, and Bayesian optimization was applied to a dataset of *in vivo* human spectra to identify key features that differentiated normal or low-cancer density tissue from dense glioma. These features included nucleic acids and protein bands, mainly collagen, phenylalanine, and tryptophan. Furthermore, features that were generated by the authors revealed important discriminating power: pairwise band interactions and interactions between patients’ age and nucleic acid bands. Importantly, they were able to quantify the uncertainty around the effect size of each feature; this is especially valuable as chemometric analysis have shown many discrepancies between different groups.

Other examples of complex analytical techniques include graph-network representations as a tool to gain insight into the data’s hidden structure, Bayesian statistics to model uncertainty in data acquisition and interpretation, and deep neural architecture to capture the complex hierarchy of the data-generating processes. Applied to RS, these emerging techniques will likely expand the yield and depth of this technology and offer richer information to researchers and clinicians.

## 7 Future Prospects in Neurosurgery and Neuroscience

### 7.1 Registering Optical Information in Neuronavigation Systems

In neurosurgery, optical measurement registration will be a critical aspect in the successful integration of Raman into the treatment protocol. Neuronavigation with infrared trackers and registration to preoperative MRI has been widely adopted by neuro-oncological surgeons. Registration of the Raman measurement to MRI images is an important aspect of future developments in intraoperative vibrational spectroscopy. The potential combination of MRI markers (such as distance to contrast enhancement, T2- or T1-weighted intensity, and apparent diffusion coefficient) with Raman-based markers could enhance the navigating environment for the operating team. In addition, MRI markers could increase the clinically relevant information content during the labeling phase of the Raman experiments. Spectroscopic measurements are meant to complement a panoply of factors that influence the extent of resection and minimize damage to normal brain; therefore, they should seamlessly integrate with the other available modalities.

In functional neurosurgery, the challenge is different, and while similar to biopsy guidance, it is more difficult due to the small target areas. In DBS, for example, as the probe descends along a planned trajectory, the position of the optical measurement must be used to update the preoperative imaging to account for any head-frame movement or brain shift.^190^ Both of these steps would require electronic drive tools to perform the physical descent to automatically correlate probe depth with the optical measurement. Although these electronic drive systems do exist for DBS surgery, manual drive screws are often used, instead, as they are faster. Since the information would ideally be merged into the current technologies that surgeons use, partnerships with commercial providers for stereotactic planning (i.e., Medtronic, Boston Scientific, Abbot) would greatly facilitate implementation in the operating room.

### 7.2 Outlook: Neurosurgery

At the present time, we may be nearing a clinical revolution in which pathology no longer requires visual confirmation by a trained clinician on site. We are seeing examples of this in the ocular industry in which AI systems for diagnosing ocular pathologies are obtaining FDA approval.^191^ Although these systems still remain tools for the physician to aid in locating abnormalities, this may not always remain the case.

In the case of neuropathology, the diagnosis could soon be achieved intraoperatively using optical techniques, greatly decreasing traditional diagnostic turnaround time. In such a situation, optical modalities will compete for a share of the biomedical market and will aim to provide the greatest advantage to the surgeon. Raman’s key advantage in this respect is the amount of information it can provide. The fingerprint type spectra can be used to quantify molecular ratios and discriminate tissue types in both a single spectrum format and in the form of content-rich hyperspectral images. Moreover, these capabilities are only beginning to be fully realized in intact brain tissue. As measurements are accumulated and sophisticated data science systems evolve for this application, a whole new Ramanomics field could emerge.^192^ To achieve this, data sharing will becoming imperative. Properly labeled raw Raman spectra databases are extremely scarce in comparison with other fields, and this is something that must change if the applied data science is to advance at a similar rate as other “omic” fields.

Although H&E stained slices are employed as “gold-standard” for labeling of Raman data, most recent guidelines on primary brain cancer classification are based on various other tissue markers such as IDH mutation status, which has proven a unique and critical factor in establishing a prognosis orienting treatment for glioma patients.^193^ Going beyond H&E staining and understanding how Raman signal changes as a function of these new biomarkers will be an important challenge of future Raman studies that aim to translate vibrational spectroscopy as a clinically valid decision-making tool.

The display of information is also an integral discussion point. In the case of Raman spectra, the raw optical data are much less important to the surgeon than what the optical information means. In the case of tissue discrimination using a point probe, a simple formulated label may suffice along with a metric for certainty. In the case of histology, SRH is already capable of displaying information in a way that would be familiar to the pathologist.^54^ Although the information may evolve to provide more than classical H&E staining, this step is absolutely mandatory for clinicians to fully understand and accept the new technology.^194^ In the case of functional surgery guidance, the live calculated position of the electrode overlaid on the preoperative MRI would likely be the ultimate goal.

### 7.3 Outlook: Neuroscience

The information that is acquired with RS is complex and difficult to interpret; therefore, there is still much to be revealed from both fundamental and clinical neuroscience research. There are a number of early stage studies showing the capacity of Raman to image or sense disease biomarkers such as prion proteins, amyloid beta plaques (Alzheimer’s), alpha synuclein (PD), and even neurotransmitters, which can be deficient in many neurological and psychiatric disorders.^51^^,^^195^^,^^196^ This work has yet to be extended to human brain tissue *in vivo*.

In the future of Raman guidance for functional neurosurgeries, we could imagine the ability to measure the relative quantities of biomarkers, and therefore the stage of the disease, to help guide treatment parameters. For instance in PD, the loss of dopaminergic neurons in the substantia nigra results in a decrease in neuromelanin.^197^ If Raman is used to guide DBS surgery in the future, it could also be used to measure either dopamine or neuromelanin concentrations to give information about the type and stage of the disease.^198^

Aging—more specifically brain-age—is another interesting topic for RS in neurosurgery. Considerable research has gone into using MRI scans to show the relation between structural changes and aging thanks to its noninvasive large-volume imaging.^199^^,^^200^ From this standpoint, Raman could be useful for complementing and understanding these observed trends, especially in the case of region-specific lipid changes associated with brain aging.^201^ Furthermore, as there is likely an age-dependent change in Raman signatures, this type of data will be critical in age-matching Raman measurements to make them even more accurate for discrimination.

Looking to the far future, if the ever-growing field of optogenetics is ever applied to humans, this would imply the implantation of chronic fiber optics within a patient. If this was to come to fruition, maintained acquisition of Raman measurements from within the brain during stimulation could be possible. Although this would either require considerable downsizing of equipment or an optical fiber port interface on the skin, this remains an enticing prospect for the future.

## 8 Conclusion

RS can provide label-free biomolecular information rapidly in a noninvasive manner and has the potential to revolutionize both neurosurgery and neurological research. The laser-based nature of Raman allows it to be incorporated into point probes, biopsy needles, and microscopes, enabling its integration into multiple points of the neurosurgical workflow. Specifically, RS has the potential to improve treatment outcomes by aiding in the detection and delineation of healthy and cancerous tissues, blood vessels, and perhaps even disease-specific biomarkers. Although this is promising, designing a Raman system for neurosurgical applications demands significant technical considerations both in terms of hardware implementation and data science methods, as is summarized in this paper. It is our hope that this resource helps guide future developments in RS systems for neurosurgery.
